# Mechanism of Action for HDAC Inhibitors—Insights from Omics Approaches

**DOI:** 10.3390/ijms20071616

**Published:** 2019-04-01

**Authors:** Wenbo Li, Zheng Sun

**Affiliations:** 1 Department of Molecular and Cellular Biology, Baylor College of Medicine, Houston, TX 77030, USA; wenbo.li@bcm.edu; 2 Department of Medicine, Baylor College of Medicine, Houston, TX 77030, USA

**Keywords:** histone deacetylase inhibitors (HDIs), epigenomics, transcriptomics, proteomics, metabolomics, chemoproteomics, cancer treatment

## Abstract

Histone deacetylase inhibitors (HDIs) are a class of prominent epigenetic drugs that are currently being tested in hundreds of clinical trials against a variety of diseases. A few compounds have already been approved for treating lymphoma or myeloma. HDIs bind to the zinc-containing catalytic domain of the histone deacetylase (HDACs) and they repress the deacetylase enzymatic activity. The broad therapeutic effect of HDIs with seemingly low toxicity is somewhat puzzling when considering that most HDIs lack strict specificity toward any individual HDAC and, even if they do, each individual HDAC has diverse functions under different physiology scenarios. Here, we review recent mechanistic studies using omics approaches, including epigenomics, transcriptomics, proteomics, metabolomics, and chemoproteomics, methods. These omics studies provide non-biased insights into the mechanism of action for HDIs.

## 1. Introduction

Human histone deacetylases (HDACs) are categorized into four classes that are based on sequence homology [1]. Class I includes HDAC1, 2, 3, and 8; class IIa includes HDAC4, 5, 7, and 9; class IIb includes HDAC6, and 10; class IV includes HDAC11; and class III includes SIRT1-7 [1]. Class I, II, and IV are canonical zinc-dependent HDACs and are simply referred to as HDACs in this review. Class III are NAD-dependent sirtuins that are more distantly related and they will not be discussed in this review. We shall keep in mind the following facts while we discuss HDACs and histone deacetylase inhibitors (HDIs). (i) HDACs deacetylate not only histones, but also non-histone proteins [2,3]. (ii) In addition to deacetylation, HDACs can also catalyze other deacylation reactions, such as demalonylation, desuccinylation, or decrotonylation [4,5,6]. (iii) HDACs have enzyme-independent functions [7,8,9,10].

HDIs block HDAC enzyme activity by binding to the zinc ion in the catalytic site, which blocks substrate access to the site [11,12]. HDIs usually consist of a zinc binding group, surface binding group, and a linker that connects the above two components and spans the hydrophobic catalytic site channel [11,12]. HDIs can be categorized into groups that are based on their chemical nature [13,14,15,16]. Hydroxymates, such as suberoylanilide hydroxamic acid (SAHA) (vorinostat), TSA (trichostatin A), LBH589 (panobinostat), and PXD101 (belinostat), are pan-HDIs that inhibit all HDACs. Short-chain fatty acids, such as VPA (valproic acid) and butyrate, inhibit class I and IIa HDACs. Benzamides, such as MS275 (entinostat), and depsipeptides, such as FK228 (romidepsin), inhibit some of the class I HDACs. Cyclic tetrapeptide, such as TPX (trapoxin), target some class I, IIa and IV HDACs. ACY-1215 (ricolinostat) is a selective inhibitor for HDAC6 [13,14,15,16].

**Figure 1:**
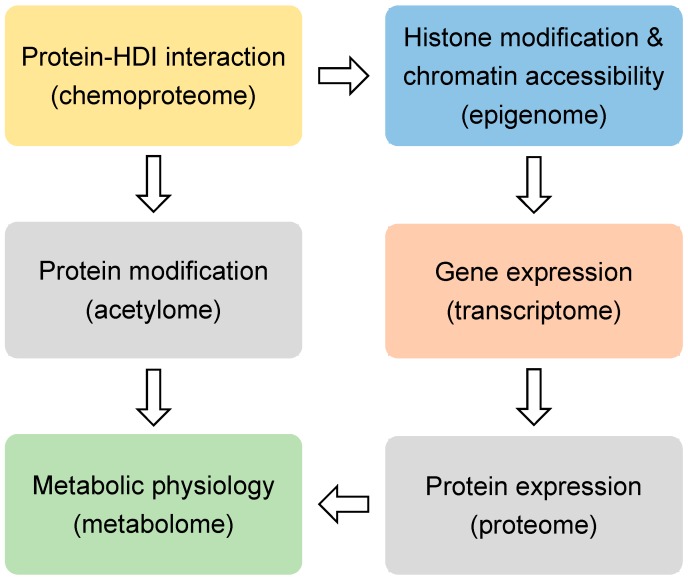
The omics approaches discussed in this review.

HDIs are being tested in over 500 clinical trials for treating many diseases, including cancers, heart diseases, diabetes, inflammatory diseases, and neurological diseases [17]. SAHA and FK228 have been approved for the treatment of cutaneous T-cell lymphoma (CTCL). PXD101 has been approved for peripheral T-cell lymphoma (PTCL), and LBH589 has been approved for multiple myeloma [18]. Most HDIs that are being tested currently are not specific to a specific HDAC. When considering that many HDACs are essential in development and physiology, such as metabolic processes [19,20], it is surprising that the nonspecific inhibition of the HDAC family could produce therapeutic effects for so many conditions. Here we review the recent non-biased studies using omics approaches (Figure 1) that provide insights into the mechanism of action for HDIs.

## 2. Epigenomic Effects of HDIs

Histones are the classical substrates for HDACs. HDIs are expected to upregulate histone acetylation levels, remodel chromatin accessibility, and upregulate gene expression. This speculation has proved to be an oversimplification by recent studies. Western blot analysis of total histone extracts can detect altered histone acetylation after HDIs treatment. However, the western blot results can be misleading, since different regions of the chromatin are differentially altered by HDIs, especially when considering that only a small fraction of the transcriptome is altered by HDIs in any given condition [21]. Chromatin immunoprecipitation (ChIP) can assess localized change of histone acetylation on the genome. Briefly, the protein-DNA complexes are cross-linked followed by fragmentation of genome DNA through sonication or enzyme digestion. Protein-bound DNA fragments are enriched with immunoprecipitation using antibodies specific for modified histones or other chromatin-binding proteins. The protein-bound genome DNA fragments are then analyzed by microarray hybridization analysis (ChIP-chip) [22] or massive parallel sequencing (ChIP-seq) [23]. Chromatin structures can range from a more open status generally associated active gene transcription to a more closed status that is associated with transcriptional silence. Open chromatin regions, which are generally enriched with regulatory cis-elements, are often free of nucleosomes and more accessible to DNA digestive enzymes or transposases. Therefore, chromatin accessibility can be assessed by DNase I, restriction enzyme (RE), or micrococcal nuclease (MNase) digestion combined with sequencing (DNase-seq or MNase-seq), formaldehyde-assisted isolation of regulatory elements (FAIRE-seq), and assay for transposase-accessible chromatin while using sequencing (ATAC-seq) [24] (Figure 1 and Table 1).

### 2.1. Epigenomic Effects of HDIs in Cancer

In the hepatocarcinoma HepG2 cells or colon adenocarcinoma HT-29 cells that were treated with butyrate or TSA, ChIP-chip analysis with antibodies against acetylated histone H3 or H4 (H3ac or H4ac) revealed that, contrary to the expected histone hyperacetylation, the histone acetylation levels were actually reduced at the transcription start sites (TSS) of actively-transcribed genes after HDI treatment. The chromatin regions with reduced acetylation levels were enriched in genes that were downregulated by the HDIs. Although histone hyperacetylation in some TSS was detected, it seemed to be transient. Trimethylation of the lysine 4 on the histone H3 (H3K4me3), a histone marker for the active promoters near TSS, was not altered by HDIs. The global histone hyperacetylation that was detected by western blot in the same study could be driven by the change in the heterochromatin regions, because the hyperacetylated regions were mainly localized in the nuclear periphery. The pan-HDI TSA showed similar H3ac and H4ac changes as the class I/IIa-selective HDI butyrate in this study [25].

In myelogenous leukemia line K562 that was treated with butyrate or SAHA, DNase I-hypersensitive (DHS) sites, as identified by DNase-seq, displayed enhancer-like properties. These regions were associated with increased gene expression and increased binding of transcription factor PU.1. However, the knockdown of PU.1 did not prevent HDIs-induced increase in accessibility and gene upregulation. The effect on the gene expression showed a high degree overlap between the pan-HDI SAHA and the class I/IIa–selective HDI butyrate [26].

The inhibition of HDAC1 and HDAC2 with small molecule ACY1035 increased the H3K27ac levels around transcription start sites, transcription termination sites, and enhancer regions in SupB15 cells, a cell model of B-cell precursor acute lymphoblastic leukemia. MNase-seq revealed that ACY1035 did not change the nucleosomal occupancy at the silent genes around transcription start and termination sites. Instead, it decreased the nucleosomal occupancy at TSSs of the expressed genes. Longtime treatment of ACY1035 (62 h) can cause even more decrease in the nucleosomal occupancies around TSS of the expressed genes. These results, together with other data in the study, suggested that the inhibition of HDAC1,2 could reduce the chromatin occupancy of chromatin remodelers and other factors that are involved in DNA repair [27]. In HT-29 cells that were treated with VPA, MNase accessibility assays found an overall more open chromatin structural state compared to non-treatment control [28].

ATAC-seq is another technique looking at chromatin accessibility. In triple-negative breast cancer (TNBC) cells HCC1806 and MDA-MB-231 treated with LBH589, a combined approach of ATAC-seq, DNA methylation BeadChip, and RNA-sequencing was used to profile genome-wide chromatin accessibility, DNA methylation, and gene expression changes [29]. No major conclusion was available from this data repository. However, these datasets could be useful in probing the mechanisms of how HDI works in breast cancer cells.

In a cutaneous T cell lymphoma (CTCL) study, an analysis of human CTCL and control samples revealed chromatin signatures that distinguished the leukemic and non-malignant T cells. SAHA or FK228 are used to treat CTCL, but only a subset (~40%) of CTCL patients respond to HDIs therapy. ATAC-seq revealed that patients with good response to HDIs showed increased DNA accessibility in CTCL cells after HDIs treatment, while patients that were resistant to HDIs did not. Surprisingly, a comparison of HDIs effects across different cell types revealed that HDIs did not open inaccessible sites in CTCL or non-malignant T cells. Rather, HDIs reinforced the existing pattern of DNA accessibility in each given cell type [30].

In melanocytes, the transition from the non-tumorigenic to tumorigenic phenotype was associated with reduced histone acetylation at specific chromatin regions, such as the promoter regions of *PI3 kinase*, *IFNγ*, *LKB1*, *TRAIL*, and *PDGF* mediated signaling. Treatment with SAHA or MS275 partially restored histone acetylation levels H2BK5ac, H4K5ac, and H3K27ac in these regions in tumorigenic cells, as revealed by ChIP-string. This could explain the tumor-specificity of the HDIs. H3K27ac ChIP-seq in five melanoma cell lines further showed that cell lines with lower histone acetylation levels were more sensitive to HDIs-induced cytotoxicity. Both the pan-HDI SAHA and the class I–selective HDI MS275 preferentially inhibited the proliferation of tumorigenic cells as compared to the non-tumorigenic cells [31].

Histone acetylation profiling reveals an effect of HDI, not the genomic location that is directly bound by an HDI. Toward the later goal, a photo-reactive probe was developed based on SAHA and it was used to identify the binding sites of SAHA on the chromatin. ChIP-qPCR with this probe in cultured MCF-7 cells revealed that SAHA bind to several promoter regions, but not the gene bodies on a canonical HDI-regulated gene *CDKN1A*, which is different from the HDAC1 ChIP pattern. These results suggest that only a subset of HDACs-bound genomic locations are targeted by HDIs. Genome-wide studies are currently lacking while using this chemical approach [32].

It is believed that histone acetylation serves as a marker for chromatin remodeling, which leads to changes of chromatin accessibility. The formaldehyde-assisted isolation of regulatory elements (FAIRE) is a chemical method for enriching the nucleosome-depleted genomic regions. Ewing sarcomas are caused by a chromosomal translocation that fuses two transcription factors—EWSR1 and FLI1—together to generate the chimeric oncoprotein EWSR1-FLI1. This fusion transcription factor is localized to specific chromatin regions and it maintains nucleosome depletion at these regions. Using a high-throughput version of FAIRE (HT-FAIRE), HDIs AR-42, SAHA, and LBH589, out of over 600 small epigenetics molecules, were identified as being sufficient in the reduction of EWSR1-FLI1-mediated chromatin accessibility. When compared to pan-HDIs, selective HDIs such as tubastatin or PCI-34051 did not alter the Ewing chromatin signature. The loss of chromatin accessibility at EWSR1-FLI1 binding sites was due to HDI-mediated alteration in EWSR1-FLI1 transcription. Therefore, HDIs suppressed the oncoprotein level, rather than directly interfering with the HDAC activity on chromatin at the target sites [33].

### 2.2. Epigenomic Effects of HDIs in the Context of other Diseases

In osteoblast MC3T3 cells, ChIP-seq with antibodies against tetraacetylated histone 4 (H4K5/K8/K12/K16ac) revealed that SAHA increased H4 acetylation across the genome. RNA-seq analysis identified that SAHA upregulated and downregulated the expression of a roughly equal number of genes (~130 in each direction). Interestingly, both the upregulated and downregulated genes were associated with H4 hyperacetylation when compared with unaltered genes, although the upregulated genes tended to have a higher level of H4 acetylation. Genes that are involved in the insulin signaling were prominently altered, which might explain why SAHA inhibited the insulin signaling and enhanced osteoblast differentiation. These data suggest that histone hyperacetylation is not sufficient for gene upregulation [34].

In primary human vascular endothelial cells HAECs that were treated with TSA as compared to non-treatment control, ChIP-seq of H3K9/14ac, H3K4me3, and H3K9me3 revealed prominent histone hypoacetylation at promoter regions near TSS, when compared to much fewer hyperacetylated promoter regions. RNA-seq analysis revealed that the hypoacetylated and hyperacetylated promoters were associated, respectively, with downregulated and upregulated gene expression in response to TSA. The hypoacetylated promoters were associated with the reduced binding of histone acetyltransferases (HATs) p300 and CBP, which could contribute to the TSA-induced histone hypoacetylation at these regions [35].

In human lymphoblastoid cells, ChIP-seq with H3K27me3 shows a strong increase in H3K27me3 at TSS of the HDIs-responsive genes, which could be altered by the inhibition of H3K27 methylating enzymes [36]. In human skeletal mesenchymal or stromal stem cells (hMSCs) that were treated with abexinostat, ChIP-seq with H3K9ac revealed that only 306 genes displayed altered acetylation levels when compared to vehicle-treated cells. Several key genes, such as *AdipoQ*, *FABP4*, and *PPARγ* that are involved in stem cell proliferation and differentiation, were among these genes [21].

In the striatum of the R6/2 transgenic mice, a Huntington’s disease mouse model, ChIP-chip showed that acetylated histone 3 (H3K9ac/K14ac) was decreased as compared to the wild-type control. This was associated with the reduced expression of genes that were bound by H3K9/K14ac at the gene body. Treatment of the transgenic mice with HDIs increased the global H3K9ac/K14ac and the expression of genes, but it only slightly increased the H3K9ac/K14ac levels at the altered genes [37].

The epigenomic effects of TSA in mouse heart *in vivo* was characterized using ChIP-seq with H3K9/K14ac in the presence or absence of the transverse aortic constriction (TAC), a pathological model that induces cardiac hypertrophy. TAC and TSA caused overall opposite effects on histone acetylation at the promoters in a gene-specific manner. TAC downregulates the H3 acetylation of genes related to metabolism and cardiac contraction, such as *Serca2a* and *Myh6*, while TSA reversed this effect by upregulating H3 acetylation at those genes. In contrast, TAC-induced hyperacetylation in genes that are involved in collagen deposition and inflammation, while TSA suppressed such effect and reduced H3 acetylation on those genes. This later effect is perplexing considering that HDAC inhibition is supposed to increase, rather than decrease, histone acetylation. It is possible that TSA may modulate acetylation and activity of NF-kB, which is a master regulator of inflammation. However, the hyperacetylation of NF-kB is supposed to enhance its activity instead of reducing it. The underlying mechanism warrants further investigation [38].

The mdx mouse is a mouse model for Duchenne muscular dystrophy. Nuclease accessibility with restriction enzyme digestion followed by sequencing (NA-seq) detected increased frequency of nuclease accessibility sites (NASs) in fibro-adipogenic progenitors (FAPs) from skeletal muscles of young mdx mice that were treated with TSA, but not old mdx mice. 70% of the detected NASs in the young mdx mice were induced *de novo* by TSA, while the rest were pre-existing NASs with accessibility levels increased after TSA treatment. Most of the altered NASs were associated with noncoding genomic sequences [39].

Another ATAC-seq study looked at the effects of short-chain fatty acids on human dendritic cells and how HDIs affect these cells’ capacity to prime and polarize T-cell responses. It found that TSA caused a stronger ATAC-seq signal in the promoter region of *RALDH1*, which was comparable to the profile that was induced by butyrate [40]. However, the pan-HDI TSA did not fully recapitulate the modulatory effects of the class I/IIa-selective HDI butyrate, particularly in terms of suppression of LPS-induced maturation marker genes. This suggests that at least some effects of butyrate are separate from its HDI activity.

In summary, the effect of HDIs on the epigenomic modification and chromatin accessibility is context-dependent. In many situations, HDIs can induce histone hypoacetylation rather than hyperacetylation, especially at promoter regions that are proximal to TSS. HDIs can increase or decrease chromatin accessibility depending on the cell, HDI, and chromatin regions being examined. The altered gene expression is generally associated with altered histone markers at the promoter regions. The pan-HDIs and class-selective HDIs tend to have common overlapping effects on global epigenomic changes, but the downstream physiological effects can be quite different.

## 3. Transcriptomic Effects of HDIs

Regardless of the upstream epigenomic mechanisms, it is generally believed that HDIs-induced changes in gene expression are ultimately responsible for their therapeutic effects in a variety of conditions. The question is what downstream gene, or network of genes, mediate the therapeutic effect of HDIs. Transcriptome profiling with microarray and RNA-seq have been performed in many studies to address this question (Figure 1). In addition to profiling the expression levels of the mature protein-coding mRNA levels, different flavors of RNA-seq techniques were also used to analyze alternative splicing forms and specific populations of RNA. miRNA-seq is specialized in detecting the small noncoding microRNA levels. Global nuclear run-on sequencing (GRO-seq) uses isolated nuclei in run-on reactions in the presence of bromouridine (BrUTP) and sarkosyl, a chemical that prevents the attachment of RNA polymerase to DNA. Thus, only pre-existing RNA polymerase on the DNA can produce new BrU-labeled transcripts, which are subsequently enriched with anti-BrU beads and analyzed by sequencing. Therefore, GRO-seq detects nascent RNA transcription (Table 1).

### 3.1. Transcriptomic Effects of HDIs in Hematopoietic and Lymphoid Cancer Cells

In acute promyelocytic leukemia NB4 cells, SAHA, in combination with retinoic acid, can overcome the transcriptional repression that is caused by the RARα fusion oncoproteins. Microarray identified genes induced by retinoic acid, SAHA, and both combined. SAHA alone upregulated 48 genes, while 80 genes were upregulated with the combination of retinoic acid and SAHA. Nine genes were found to be upregulated two to three-fold by the combination of retinoic acid and SAHA, but not induced by either drug alone. Northern blot analysis confirmed that the transcription levels of *transglutaminase 2* and *p21^WAF1^* were higher after the combined treatment when compared to any one drug alone. These genes may contribute to the synergistic effects of SAHA and retinoic acid in treating acute promyelocytic leukemia [41].

Microarray analysis in leukemic cell lines CCRF-CEM and HL-60 found that a large number of genes (9% of the genome) were similarly regulated in both cell lines in response to TSA. Temporal analysis of gene expression distinguished primary and secondary responses. Many of the HDIs-altered genes encode trans-acting factors that are highly relevant to cancer [42]. An imprinted gene *MKRN3* was the most rapidly repressed gene and was involved in the Prader–Willi syndrome [42]. In acute myeloid leukemia (AML) MV-4-11 cells, microarray analysis identified respective and overlapping genes altered by an HDAC1/2 inhibitor ACY1035 and a DNA methyltransferase inhibitor azacitidine [43].

In another study with human promyelocytic leukemia HL60 cells that were treated with VPA, SAHA, and TSA, microarray analysis found that only ~9% of the detected genes showed altered expression levels after treatment. SAHA altered 5% of the detected genes, and TSA altered 13%, with roughly half being downregulated and half upregulated in each case. However, the responsive genes to each HDI were unique. More than 67% of the responsive genes of each HDI were not regulated by the other two HDIs. Most of the upregulated genes showed minimal change in histone acetylation at the promoter regions [44].

High-density microarray analysis was carried out to describe the dynamic changes in the transcript levels in the human lymphoblastoid cell lines GM12878 and AH-LCL when treated with SAHA and VPA. About 7% of the genes showed significant expression change. For each HDI, the responsive genes at different concentrations had strong overlap. SAHA altered 73% of the VPA-responsive genes. These genes were highly enriched in transcriptional regulation, including many DNA-binding zinc-finger proteins. HDIs downregulated histone acetyltransferases (HATs). *GDF9*, a TGF-β superfamily gene essential in G1-S and G2-M progression, and its paralogue *GDF15* were downregulated by all HDIs. The transcriptional response to HDIs was associated with the H3K27me3 changes at the TSS of these genes [36].

In primary T cells that were treated with TSA, microarray analysis revealed that, out of 2352 detected genes, 48 genes showed significant and reproducible RNA expression changes. Some costimulatory or adhesion genes, such as *CD28* and *CD154*, were downregulated with TSA treatment. This indicated that HDI might have immunomodulatory potential [45].

In T cell leukemia CEM cells that were treated with SAHA or FK228, microarray analysis revealed that 22% and 24% of the detectable genes showed altered expression. Both SAHA and FK228 regulated genes involved in apoptosis and cell cycle regulation. Signaling pathways related to *Myc*, *type β TGF*, *cyclin/cyclin-depend kinase*, *TNF*, *Bcl-2* and caspase pathways were regulated by HDIs toward the induction of apoptosis and decrease of cellular proliferation. Both SAHA and FK228 induced the expression of *APAF-1*, which is an intrinsic apoptotic pathway gene. The transcriptomic changes that are induced by the pan-HDI SAHA highly overlapped with that by the class I-selective HDI FK228 within the first 4h of treatment. However, the responses diverged as time progressed [46].

In cutaneous T-cell lymphoma (CTCL) cells that were treated with SAHA or LBH589, miRNA microarray identified that 161 miRNAs were commonly upregulated by both HDIs. Among them, miR-150 was known to have a tumor suppressive function. Further cellular assay and animal experiments confirmed that miR-150 could efficiently inhibit CTCL migration and metastasis [47].

In biopsy tissues of human CTCL that were treated with LBH589, microarray analysis showed rapid gene expression change. Among the altered genes, LBH589 downregulated more genes than it upregulated. The expression change of 23 genes was similar in all of the patients tested. These common genes were involved in apoptosis, immune regulation, and angiogenesis. These genes could serve as potential molecular biomarkers for HDI activity in CTCL cells [48].

In B cells that were stimulated by LPS plus IL-4 and treated with VPA, RNA-seq analysis showed that VPA downregulated *Aicda* and *Prdm1*, while miRNA-seq analysis showed that VPA upregulated miR-23b, miR-30a, and miR-125b. *Aicda* is a target of miR-125b, whereas *Prdm1* is a target of miR-23b, miR-30a, and miR-125b. Only 0.36% of the high copy number genes were downregulated by VPA by 50% or more. Only 0.30% of the highly expressed genes were upregulated by more than two-fold by VPA [49]. In SK-MEL-3 melanoma cells that were treated with TSA, RNA-seq revealed the altered genes were enriched in the MAPK signaling pathway, cell-cycle regulation, and apoptosis [50].

Single-cell RNA-seq (scRNA-seq) of tumor-infiltrating T cells from mice harboring *Kras^G12D^* concurrent with *P53* loss (KP) treated with ACY-1215 showed upregulation of T-cell activation and T-cell receptor signaling genes, including *Cd69*, *Cd44*, *Cd247,* and *Zap70*. Single-cell RNA-seq of tumor-associated macrophages among myeloid cell populations, showed that ACY-1215 treatment induced the expression of key genes related to MHC class II expression, such as *Cd74* and *H2-Aa*, when comparing to vehicle treatment in genetically engineered mouse models of non–small cell lung cancer [51].

### 3.2. Transcriptomic Effects of HDIs in other Cancer Cells

In lung cancer cells that were treated with a novel HDAC inhibitor TMU-35435, microarray analysis identified cell proliferation and the Wnt signaling pathway as enriched biological processes [52]. In H460 human lung carcinoma cells that were treated with butyrate, microarray analysis revealed 32 genes with greater than two-fold increase and 66 genes less than one-third expression level as compared with the untreated cells. Among the downregulated genes, 16 were associated with cytokine signaling pathway. Butyrate also upregulated three metastatic suppressors and downregulated 11 metastasis activation genes. These genes could contribute to the anti-inflammatory and anti-metastatic effects of butyrate [53].

In one study, selective HDAC6 Inhibitor ACY-1215 and/or BET inhibitor JQ1, as well as vehicle control, were applied to athymic nude mice carrying human small cell lung cancer (SCLC) NCI-H69 xenograft tumors. A gene expression heat map was generated from RNA-seq analysis of these xenograft tumors, which showed that ACY-1215 treatment had limited change with transcription profiling as compared to JQ1 treatment [54].

In esophageal cancer cells that were treated with FK228, microarray analysis showed that 93 and 65 genes in human esophageal cancer T.Tn and TE2 cells had more than two-fold expression level change when treated with FK228. The expression change patterns of 15 genes were similar between these two cell lines. Some of these genes, such as *p21^WAF1^* and *Prdx*, are involved in cell cycle arrest or tumor suppression [55].

In prostate cancer LNCaP cells that were treated with two hydroxamic acid-based HDIs, TSA and CG-1521, microarray showed differential gene expression patterns. CG-1521 regulated a very limited number of genes that were involved in cell cycle progression and cell death, while TSA induced much more genes that did not overlap much from those genes that were altered by CG-1521 [56].

In colon cancer HCT116 and HT29 cells that were treated with SAHA and LBH589, microarray analysis showed similarity in terms of the differentially expressed genes that were induced by the two HDIs within each cell line. However, the HCT116 and HT29 cells showed differential responses to HDI treatment. Only 11 genes were modulated in both cell lines with both HDIs in a similar manner [57].

In cervical cancer HeLa cells treated with butyrate, splicing-sensitive microarrays identified about 700 genes with altered RNA splicing upon butyrate treatment. By measuring different pre-mRNA levels along the *FN1* gene using polymerase II ChIP assay, the authors showed that butyrate also increased the processivity of RNA polymerase II along an alternatively spliced element. The depletion of HDAC1 had similar effect as butyrate. This effect was reversed by a wild-type, but not catalytically inactive HDAC1 mutant, suggesting that the effect was mainly mediated by the inhibition of HDAC1 [58]. In bovine epithelial cells, RNA-seq analysis with a focus on alternative splicing event revealed that butyrate induced more than 2,000 exon-skipping events. Some of the events occurred on genes with altered expression levels upon butyrate treatment. In addition, butyrate also affected 13 gene fusion events [59].

In breast cancer MCF-7TN-R cells, miRNA microarray analysis revealed that TSA upregulated 22 miRNAs and downregulated 10 miRNAs [60]. In another study with MCF-7 cells that were treated with TSA, a targeted-transcriptome profiling technique, named templated oligo assay with sequencing (TempO-seq), found more than 9000 differentially expressed genes when compared to vehicle control. Comparing the TSA-induced transcriptomic change profiles in HL-60 cells and PC-3 cells revealed around 1000 differentially expressed genes in common, which could serve as a cell-type-independent TSA signature [61]. In MDA-MB-231 breast cancer cells that were treated with the benzoylhydrazide analog UF010 and vehicle, microarray analysis found that UF010 altered the expression of many genes that are involved in cell cycle regulation. UF010 also upregulated *histocompatibility complex* (*MHC*) and immune response genes [62].

Global run-on sequencing (GRO-seq) was used to measure nascent transcription in a breast cancer cell line BT474 and a non-cancerous breast epithelial cell line MCF10A. TSA was found to repress transcription by blocking RNA polymerase II elongation. TSA preferentially downregulated the transcription of the highly expressed genes, as well as high copy number genes in the cancer cell line genome. In contrast, genes that were upregulated by TSA were likely to be moderately expressed. The downregulation of amplified oncogenes might explain the cancer-specific lethality of HDI [63].

In synovial sarcoma cells treated with class I and II HDI quisinostat, RNA-seq showed that the genes with altered expression were enriched in cell-cycle arrest, neuronal differentiation, and cellular response to reactive oxygen species. In particular, many pro-apoptotic factors, such as *BCL2L11*, *BIK*, and *BMF*, were upregulated by quisinostat [64].

In the thyroid cancer cell lines, microarray identified that PXD101 and LBH589 altered the expression of 528 and 573 genes, respectively, with 153 genes being commonly altered by both HDIs. The top biological pathways of the 153 genes were cell cycle regulation, DNA damage, and apoptosis [65]. In adrenocorticotropic hormone (ACTH)-secreting pituitary tumor AtT-20 cells, microarray analysis identified that SAHA downregulated several nuclear receptors, including LXRα, and upregulated many genes of the mitochondria-mediated cell death pathway. Interestingly, SAHA-mediated downregulation of *LXRα* was not observed in normal corticotrophs [66].

In pancreatic ductal adenocarcinoma (PDAC) cell lines that were treated with a class I-specific HDI domatinostat (4SC-202), RNA-seq and H3K27ac ChIP-seq revealed that the HDI-upregulated genes were associated with increased H3K27ac at the regions proximal to TSS. The ReMap (regulatory map of TF binding sites) analysis tool [87] showed an enrichment of BRD4 and MYC binding sites in these regions as compared to genes that were not altered or downregulated by HDI. Further knockdown experiments confirmed that BRD4 and MYC were indeed required for the HDI-induced upregulation of many genes. When compared to HDI-upregulated genes, the HDI-downregulated genes were associated with decreased H3K27ac in regions that were more distal to TSS. GREAT analysis [88] on these genomic regions implicated a few genes, including *SMAD6* and *E2F8*, in the process [67].

In brain tumor LN-18 cells, RNA-seq revealed that SAHA upregulated around 4000 genes and downregulated around 3500 genes. A combination of SAHA treatment with the knockdown of *lysine-specific demethylase 1* (*KDM1A*) led to altered expression of around 1000 genes that were not changed by either treatment alone. 179 genes were common among all treatment regimens. Apoptosis-related genes were enriched among these common genes. Tumor suppressor genes *TP53* and its paralog *TP73* were decreased by the combined treatment, which may contribute to the cell death in response to the combined treatment [68].

### 3.3. Transcriptomic Effects of HDIs in Chronic or Degenerative Diseases

In addition to the anti-cancer effect, transcriptomic profiling has been used to explore the other therapeutic or toxic effects of HDIs in vivo or in cultured cells. Microarray analysis identified 655 genes with altered expression in the brain, cerebellum, or heart of either KIKI mice (a genetic mouse model for Friedreich ataxia) or wild-type mice when treated with compound 106, an HDI that inhibits class I HDACs. The overall effect of HDI was limited. The apoptosis and the tumorigenesis-related genes were not significantly changed. KIKI and WT mice had a set of genes with different expression levels without any treatment. Among these genes, 67% in brain, 84% in cerebellum, and 67% in heart showed changes toward the normal levels after the treatment of compound 106 in KIKI mice. In WT mice, the same group of genes showed no significant change or only minor changes after the treatment of compound 106. These findings indicated that the gene expression difference in KIKI mice as compared to WT mice could be partially reversed by compound 106, which may contribute to the therapeutic effect of HDI against Friedreich ataxia [69].

Increased H3 acetylation level in the nucleus accumbens in mice with chronic social defeat stress is associated with a decreased level of HDAC2. Infusion of MS275 reversed the global gene expression pattern in the nucleus accumbens of mice with chronic defeat stress. Microarray analysis revealed overlap effects of MS-275 with fluoxetine, an antidepressant medication. MS275 in mice with chronic social defeat upregulated about 37% of the fluoxetine-upregulated genes. These genes may underlie the therapeutic effect of HDI against social defeat stress [70].

In bone marrow derived macrophages (BMDMs), microarray analysis showed that TSA downregulated innate immune genes, which is in line with the TSA-mediated inhibition of microbe-induced cytokine release. This could be contributed by the upregulated expression level and DNA binding activity of the transcriptional repressor Mi-2β [71]. HDIs could also help to regenerate dental pulp tissue. In dental pulp cells (DPCs) that were treated with SAHA, microarray identified around 1100 altered genes [72].

To investigate the teratogenicity of VPA and its analogs, microarray analysis was performed on undifferentiated R1 mouse embryonic stem cells that were exposed to nonteratogenic analog 2-ethyl-4-methylpentanoic acid (2-Et-4-Me-Penta), VPA, and the teratogenic VPA analog (S)-2-pentyl-4-pentynoic acid (S-2-Pe-4-Pentyn) for six hours. VPA and S-2-Pe-4-Pentyn regulated much fewer genes compared to 2-Et-4-Me-Penta. The expression pattern of five genes was sufficient to separate the teratogens from nonteratogens. Many teratogenicity related genes were involved in embryonic development and morphogenesis, including neural tube formation and closure [73].

In an age-associated memory impairment mouse model treated with SAHA, RNA-seq of the hippocampal CA1 region showed that, out of 1,973 differentially expressed genes in old mice when compared to young mice, 83% could be reversed by SAHA administration. This reverse effect can be observed in both up- and downregulated genes. In agreement with the RNA-seq analysis, H4K12ac ChIP-seq showed a decreased H4K12ac level around TSS in the neuronal cell population in old mice, which can be reinstated by SAHA. The non-neuronal cells showed similar H4K12ac levels around TSS between young and old mice. In contrast, RNA-seq analysis of the liver samples showed that the transcriptomic difference between old mice and young mice is highly enriched with genes involved in inflammation. In addition, SAHA treatment did not normalize the gene expression change in the liver of old mice. This age-reversal effect of SAHA on gene expression and histone acetylation specifically in neuronal cells may explain the cognitive improvement in aged mice after SAHA treatment [74].

In mouse brain and muscle samples that were treated with an HDI, HDACi-4b, microarray analysis revealed that the biological pathway involving DNA methylation was enriched. Follow-up DNA methylation analysis in human fibroblast identified some methylated CpG sites that were altered by the HDI. HDI increased the methylation of *lysine-specific demethylase 5D* (*KDM5D*), and further animal studies suggest that HDI can induce transgenerational effects through such alteration of DNA methylation [75].

In fibro-adipogenic progenitors (FAPs) from young mdx mice that were treated with TSA, microarray analysis found that the TSA-upregulated genes were involved in muscle determination, differentiation, muscle contraction, and metabolism. TSA induced the expression of muscle-specific determination factor MyoD and chromatin remodeling complex subunit BAF60C. High-throughput screening of miRNAs (miRs-HTS) and small RNA-seq identified that TSA upregulated the myogenic miRNAs, including miR-1.2, miR-133, and miR-206 [39].

In summary, HDIs change the expression of genes that are involved in cell proliferation, cell death, stress response, and DNA damage in many cancer cells, which may underlie their anti-cancer effects. The transcriptomic effects of HDIs are dependent on the cell and tissue. There is no consensus regarding what downstream target genes mediate the therapeutic or toxic effect of HDIs in each context given the general lack of the follow-up functional analyses in the profiling studies. There are generally distinct transcriptomic signatures between pan-HDIs and selective HDIs, although overlapping effects were observed under some conditions.

## 4. Proteomic and Acetylomic Analysis of HDIs

It is conceivable that the epigenomic and transcriptomic changes elicited by HDIs need to be ultimately manifested at the protein level for the downstream target genes in order to have a physiological effect (Figure 1). The mass spectrometry (MS)-based approaches have been used to profile the proteomic changes that were caused by HDIs in several studies. Non-labeling and a variety of labeling methods have been developed for quantification. Stable isotope labeling by amino acids in cell culture (SILAC) uses cell culture medium that contains amino acids labeled with stable heavy isotopes. When combined with non-labeled cells for MS analysis, chemically identical peptides with different isotope composition can be distinguished, and their relative abundance reflects the relative abundance of the two proteins [89]. This method allows for a more accurate quantification when compared to the non-labeling methods. In addition to histone acetylation, HDIs may affect acetylation of many non-histone proteins, including transcription factors and co-factors [90,91,92]. The acetylome approach uses immunoprecipitation with pan-anti-acetylated lysine antibodies to enrich the acetylated peptides from the total peptides of a biological sample. The subsequent MS analysis allows for comprehensive detection and quantification of acetylated proteins [93] (Table 1).

Impaired cholesterol efflux from the late endosomal/lysosomal (LE/L) compartment characterizes Niemann–Pick type C (NPC) disease. SAHA can restore the cholesterol level in NPC patient-derived NPC1^I1061T^ fibroblast cells. An isobaric labeling-based quantitative proteomic profiling of fibroblasts identified a total of 202 proteins differentially expressed in SAHA-treated fibroblasts versus vehicle control. Gene ontology and pathway analysis showed that most of the 202 genes were mapped to metabolic pathways, and about one-third of the genes were mitochondria-associated. 132 proteins were upregulated, while 70 were downregulated after SAHA treatment. Lysosomal acid lipase (LIPA) was particularly interesting, because it played an essential role in mediating cholesterol efflux in NPC^I1061T^ fibroblasts [76].

In esophageal squamous cell carcinoma EC109 cells that were treated with FK228, SILAC-based proteomic analysis identified that FK228 altered acetylation levels on 87 lysine sites from histone and altered the protein levels of 3,515 proteins. Among the 3515 proteins, 675 proteins showed an upregulation of lysine acetylation levels, and 186 proteins showed downregulated acetylation levels. Gene ontology (GO) analysis revealed that the many proteins involved in type I interferon signaling pathway and the protein stability regulation were upregulated by FK228, whereas those that were associated with intermediary metabolism and hormone biosynthesis were downregulated. Enrichment analysis of the molecular functions revealed that the actin-binding and metal-binding proteins were upregulated. Proteins related to oxidoreductase or aldo–ketoreductase activities were downregulated [77].

In acute myeloid leukemia (AML) HL60 cells that were treated with SAHA and VPA, a combination of SILAC, anti-acetyl-lysine affinity enrichment, and liquid chromatography-tandem mass spectrometry (LC-MS/MS) approaches identified 5775 proteins and 1124 lysine acetylation sites as responsive to SAHA or VPA treatment. SAHA upregulated 323 proteins and downregulated 452 proteins, whereas VPA upregulated 359 proteins and downregulated 426 proteins. GO-based classification showed similar distribution between SAHA and VPA regarding the ontology of biological process, cellular component, and molecular function. Molecular function-based clustering showed that VPA upregulated proteins involved in the phosphatidylinositol phosphate phosphatase activity and cargo receptor activity, whereas it downregulated the proteins that were involved in cysteine-type endopeptidase inhibitor activity, phosphoprotein binding, and isocitrate dehydrogenase activity. In the cellular component category, VPA upregulated proteins that were involved in plasma lipoprotein particle, and transcription regulation related protein complexes, such BAF and SWI/SNF complexes, while SAHA upregulated proteins involved in vesicular transportation and transmembrane transportation. A clustering analysis based on KEGG pathway showed that leukocyte trans-endothelial migration, lysosome, platelet activation, and apoptosis were the dominant pathways that were enriched in the upregulated proteins by both VPA and SAHA, while many amino acid metabolism pathways were enriched in the downregulated proteins by both VPA and SAHA. For acetylome profiling, VPA upregulated acetylation levels on 186 lysine sites of 164 proteins and downregulated acetylation levels on 135 lysine sites of 104 proteins. SAHA upregulated acetylation levels on 139 sites of 124 proteins and downregulated 94 sites of 88 proteins. Proteins with differential acetylation levels upon VPA and SAHA treatment show similar distribution based on GO and subcellular location analysis. Protein–protein interaction network analysis revealed that top nodes in HDIs-induced interactome were heat shock proteins, protein chaperones, ribosome proteins, ATP-citrate synthase, and small nuclear ribonucleoproteins [78].

In HeLa cells that were treated with 19 different HDIs, SILAC based quantitative mass spectrometry was used to profile acetylation change. For all HDIs except sirtinol, more lysine sites showed upregulated acetylation levels than the sites with downregulated acetylation levels. Interestingly, most HDIs increased the acetylation level above the threshold on only a small fraction (average 6.2%) of the quantified sites, including sites on histones and non-histone proteins. Genetic studies showed that nicotinamide worked through the inhibition of SIRT1, while tubacin and bufexamac worked through inhibition of HDAC6 [79].

In summary, HDIs not only alter the proteomic profile, they also alter the acetylomic profile on histone and non-histone proteins. HDI-altered proteins were enriched in metabolism, immune responses, intracellular protein trafficking, and protein quality control.

## 5. Metabolomic Effects of HDIs

Metabolic changes are recognized as a hallmark of cancer [94]. Many HDACs are known to regulate the metabolism of nucleotides, lipids, carbohydrates, amino acids, and other intermediates [95,96,97] (Figure 1). With the recent development of the nuclear magnetic resonance (NMR) or MS-based metabolomics methods, profiling of the metabolic changes in response to HDIs has been performed. In addition to the static snapshot profiling, stable-isotope tracers can also be used to analyze the kinetic flux through a variety of metabolic pathways, an approach that is sometimes referred to as fluxomics [98] (Table 1).

To characterize the role of HDIs in cell differentiation, a tracer-based metabolomics approach was carried out in human colorectal adenocarcinoma HT29 cells with the treatment of butyrate or TSA. [1,2-^13^C_2_]-d-glucose was used as a tracer to measure the metabolic flux through glycolysis, the pentose phosphate pathway (PPP), and the Krebs cycle. Butyrate and TSA decreased glycolysis, the use of the oxidative branch of the PPP, and pyruvate dehydrogenase contribution. TSA and butyrate induced similar metabolic profiles and induced the differentiation of HT29 cells, which was different from the metabolic effects of acetyl-CoA precursors. When considering that butyrate itself is an endogenous precursor for acetyl-CoA, these data suggest that the metabolic change of butyrate is mainly due to its HDI activity, rather than a precursor of acetyl-CoA. The study also suggests a potential connection between HDIs-induced metabolic changes and cell differentiation [80].

Proton nuclear magnetic resonance (^1^H-NMR) metabolomics analysis was performed in glioblastoma U373 and LN229 cell lines that were treated with SAHA, TSA, HDACIIb inhibitor tubastatin A (TUBA), some novel HDIs (i8, i10, or i12), or the SIRT inhibitor nicotinamide (NAM). Both the intracellular and extracellular metabolites were measured. The intracellular metabolites showed a distinct pattern between U373 and LN229 cells. The LN229 cells showed major metabolic changes in cellular metabolism and metabolite transport upon treatment with i8, i10, i12, SAHA, or TUBA. LN229 cells that were treated with SAHA showed drastic differences in intracellular profiles, including elevated levels of lactate, glucose, and TCA cycle intermediates. The treatment of LN229 cells with i8, i10, or i12 also changed choline-related metabolites. However, for the intracellular metabolites from U373, only isocitrate showed significant changes, suggesting that metabolic profiles inside of the U373 cells were relatively unaffected by SAHA. Interestingly, HDIs altered the extracellular metabolites from the U373 cell culture medium, which suggests an increase of metabolite transport to or from the extracellular medium. These findings showcased the heterogeneous responses to HDIs among the different cell lines [81].

To determine the metabolic effects of HDIs on diffuse large B-cell lymphoma (DLBCL), untargeted MS-based metabolomic analysis was performed on plasma samples from patients that were treated with LBH589. LBH589 upregulated several sulfated steroids and bile acids in a xenobiotic metabolism pathway. LBH589 downregulated metabolites branched chain amino acids (BCAA) and betaine, which is an atypical amino acid derived from the oxidation of choline. The further metabolomic analysis in DLBCL cell lines OCI-Ly1 and OCI-Ly7 revealed that LBH589 upregulated many metabolites in the betaine-choline pathway, including choline, cytidine diphosphate, CDP-choline, and 1-oleoylglycerophosphocholine. Gene expression analysis revealed the upregulation of choline kinase α (CHKA) and phosphocholine cytidylyltransferase (PCYT1A) in DLBCL cell lines that were treated with LBH589. Further functional study regarding CHDA inhibition or knockdown demonstrated that targeting the choline pathway increased the anti-cancer effect of HDIs in DLBCL [82].

In cultured pancreatic cancer BxPC3 cells and hepatocellular cancer Hep1 cells, ^1^H-, ^13^C-, and ^31^P-NMR spectroscopy with [1–^13^C]-glucose tracer did not identify any prominent changes in cell metabolism after treatment with PXD101 [99]. In rats that were treated with HDIs, gas chromatography-mass spectrometry analysis of the serum revealed that SAHA upregulated urea, oleic acid, and glutaconic acid, whereas it downregulated octadecanoic acid, pentadecanoic acid, glycerol, propanoic acid, and uric acid [100]. MGCD0103, another HDI, downregulated alanine, isoleucine, and leucine levels in the serum [101].

In summary, HDIs can alter the metabolism of amino acids, choline, or carbohydrates, which may contribute to their therapeutic or toxic effects in different tissues.

## 6. Chemoproteomic Analysis of HDIs

Regardless of the downstream epigenomic, transcriptomic, proteomic, or metabolomic changes that HDIs may cause, it is conceivable that HDIs must have physical interactions with their direct targets. Small molecules are notoriously promiscuous, and it is possible that HDIs could bind to many proteins and alter their functionality, in addition to HDACs. Chemoproteomic approaches with chemical probes allow the systemic identification of proteins bound to a small molecule. These methods have been used to profile HDI-interacting proteins in several studies (Table 1).

The HDIs affinity probes were synthesized by conjugating sepharose to analogs of SAHA or class I and class II-selective HDI givinostat. After exposing the probe with protein extracts from the myelogenous leukemia K562 cells, the captured proteins were quantified with mass spectrometry. Competition analysis with HDIs was then performed to reduce the binding affinity of each HDI with the identified binding proteins, generating a dataset composed of 16 HDIs with over 1000 proteins. Immunofluorescence analysis of acetylated histones or tubulin further validated the range of activity for several HDIs, as determined in the chemoproteomic analysis. Class IIa HDACs were not identified in the assay, which is consistent with their low affinity to hydroxamate and lack of catalytic activities due to a missense mutation in the catalytic pocket [102]. The anti-inflammatory drug bufexamac was identified as a novel HDI targeting class IIb HDACs. The approach also identified the interaction between some HDIs with many non-HDAC proteins that do not appear to be in any HDAC protein complexes [83]. The chemoproteomics-based competition analysis with the same chemical probe was later used to characterize the binding kinetics property of different HDIs with endogenous protein lysates from K562 cells [84]. As is consistent with the previous assays with recombinant HDAC proteins [12], aminobenzamides showed slower binding kinetics with HDACs-containing protein complexes when compared to hydroxamates [84].

A similar approach from a separate study with SAHA-coated sepharose beads and HPLC-MS/MS identified 58 proteins as potential SAHA binding proteins in the HeLa cell lysates. 35 proteins were found as HDAC complex components. Gene ontology analysis showed that 23 proteins were metabolic enzymes, four proteins were involved in cell cycle regulation and differentiation, and two proteins were chaperones. A glucose metabolism enzyme ENO-1 was identified as a novel SAHA-binding protein and may be a target of HDIs [85].

Another study used the chemoproteomic approach to explore the mechanism of the 2-aminobenzamide class of HDIs in treating Friedreich’s ataxia (FRDA) and Huntington’s disease. A chemical probe was synthesized by connecting the HDI compound 106 with two structures: one to form a covalent bond with target proteins upon UV crosslink and another to be conjugated with biotin through click chemistry. This active probe and a control probe were incubated with nuclear protein extracts from FRDA patient-derived neural stem cells. After UV crosslink and biotin conjugation, a quantitative proteomic method using multidimensional protein identification technology (MudPIT) was used to identify the proteins that were specifically captured by the probe. Among the 3003 detected proteins, 883 were enriched more than two-fold with the active probe as compared to the control probe. HDAC1, HDAC2, and HDAC3 showed enrichment, which was confirmed by western blot. Gene ontology analysis showed that the active probe-binding proteins were highly enriched in the translation elongation and ribosome-associated pathways. This finding suggests that RNA processing and translational regulation may contribute to HDIs-mediated therapeutic effect in FRDA [86].

In summary, HDIs can interact with a variety of protein and protein complexes, including those that are not directly associated with HDACs. Their profile of interacting proteins tends to be different for different HDIs.

## 7. Conclusions

Multiple omics approaches have started to shed light on the mechanisms that underlie therapeutic or toxic effects of HDIs, although the mechanism is likely context-dependent and there is no consensus mechanism on the horizon. Although HDIs can upregulate global histone acetylation levels, HDIs do not always cause histone hyperacetylation, especially at promoter regions near gene transcription start sites. HDIs tend to upregulate and downregulate an equal number of genes, although the relative ratio is dependent on the binding kinetics of the HDIs and the timing of the assay. HDIs regulate the expression of genes that are involved in diverse biological pathways, including cell cycle regulation, cell death, metabolism, and stress responses in many cancer cells. The changes in histone acetylation levels at gene promoters tend to correlate with the changes in gene expression levels in most cases, although the gene expression changes are not always associated with phenotypic changes. In addition to epigenomic and transcriptomic profiling, recently developed proteomics, metabolomics, and chemoproteomics approaches have started to provide multifaceted datasets regarding HDIs. Pan-HDIs tend to have distinct effects from class-selective HDIs in general, although overlapping effects were observed under some conditions. The availability of these omics datasets provides opportunities for data mining with artificial intelligence. For example, the correlation of differential responses to HDIs among cancer cell lines with transcriptomic and proteomic effects of HDIs on these cells could shed light on the downstream targets that mediate certain cellular responses to HDIs. Continuous application of these omics measurements and integrated bioinformatics analysis of the datasets shall provide novel mechanistic insights into the HDIs-mediated biological effects.

## Figures and Tables

**Table 1:** Epigenomics (blue), transcriptomics (orange), proteomics (gray), metabolics (green), and chemoproteomics (yellow) studies of histone deacetylase inhibitors (HDIs). Cells are from human origin unless otherwise indicated.

Analysis	HDI	Cell or Tissue	Reference
H3 and H4 ChIP-chip	butyrate and TSA	hepatocarcinoma HepG2 cells and colon adenocarcinoma HT-29 cells	[25]
DNase-seq	butyrate and SAHA	K562 myelogenous leukemia cells	[26]
MNase-seq	ACY1035	BCR-ABL1-expressing leukemic cells SupB15	[27]
MNase-seq	VPA	HT-29 colon cancer cells	[28]
ATAC-seq, DNA methylation BeadChip and RNA-sequencing	LBH589	triple-negative breast cancer cell lines HCC1806 and MDA-MB-231	[29]
ATAC-seq	SAHA or FK228	cutaneous T cell lymphoma tissue	[30]
ChIP-seq	SAHA and MS275	human primary foreskin melanocytes HMEL-BRAFV600E and PMEL-BRAFV600E cells	[31]
ChIP-qPCR	SAHA	MCF-7 breast cancer cells	[32]
HT-FAIRE	AR-42, SAHA, and LBH589	Ewing sarcoma patient-derived EWS894 and EWS502 cells	[33]
H4K5/K8/K12/K16ac ChIP-seq and RNA-seq	SAHA	osteoblast MC3T3 cells	[34]
ChIP-seq, MBD-seq and RNA-seq	TSA and SAHA	primary vascular endothelial cells HAECs	[35]
H4K16/H3K9/K27ac and H3K27me3 ChIP-seq	VPA and SAHA	GM12878 and AH-LCL lymphoblastoid cells	[36]
H3K9Ac ChIP-seq and microarray	abexinostat	mesenchymal or stromal stem cells (hMSCs)	[21]
H3K9ac/K14ac ChIP-chip	phenylbutyrate	wild-type and R6/2 mouse liver	[37]
H3K9/K14ac ChIP-seq	TSA	mouse heart tissue	[38]
Nuclease accessibility sequencing (NA-seq)	TSA	fibro-adipogenic progenitors (FAPs) from mdx mice	[39]
ATAC-seq	TSA and butyrate	dendritic cells	[40]
microarray	SAHA	acute promyelocytic leukemia NB4 cells	[41]
microarray	TSA	T lymphoblastoid leukemic CCRF-CEM cells and promyelocytic HL-60 cells	[42]
microarray	ACY1035	MV-4-11 acute myeloid leukemia cells	[43]
microarray	VPA, SAHA and TSA	promyelocytic leukemia HL60 cells	[44]
microarray	VPA and SAHA	GM12878and AH-LCL lymphoblastoid cells	[36]
microarray	TSA	primary T cells	[45]
microarray	SAHA and FK228	acute T cell leukemia cell line CEM cells	[46]
miRNA microarray	SAHA and LBH589	My-La, HH, and HUT78 cutaneous T-cell lymphoma cells	[47]
microarray	LBH589	cutaneous T-cell lymphoma tissue	[48]
miRNA-seq and mRNA-seq	VPA	mouse B cells	[49]
RNA-seq	TSA	SK-MEL-3 melanoma Cells	[50]
single-cell RNA-seq	ACY-1215	tumor-infiltrating T cells from mice harboring Kras^G12D^ concurrent with P53 loss (KP) and tumor-associated macrophages among myeloid cell populations	[51]
microarray	TMU-35435	MRC5 and IMR90 lung cancer cells	[52]
microarray	butyrate	H460 lung cancer cells	[53]
RNA-seq	ACY-1215	SCLC NCI-H69 xenograft tumors carried by athymic nude mice	[54]
microarray	FK228	esophageal cancer cell lines T.Tn and TE2	[55]
microarray	TSA and CG-1521	LNCaP prostate cancer cells	[56]
microarray	SAHA and LBH589	HCT116 and HT29 colon cancer cells	[57]
splicing-sensitive microarray	butyrate	HeLa cervical cancer cells	[58]
RNA-seq	butyrate	bovine epithelial cells	[59]
miRNA microarray	TSA	MCF-7TN-R breast cancer cells	[60]
TempO-seq	TSA	MCF-7 breast cancer cells	[61]
microarray	UF010	MDA-MB-231 triple-negative breast cancer cells	[62]
GRO-seq	TSA	BT474 breast cancer cells	[63]
RNA-seq	quisinostat	SYO-q, FUJI, YaFuss, HS-SY-II, MoJo, Yamato-SS synovial sarcoma cells	[64]
microarray	PXD101 and LBH589	BHP2-7 thyroid cancer cells	[65]
microarray	SAHA	ACTH-secreting tumor cells AtT-20	[66]
RNA-seq and ChIP-seq	FK228	pancreatic ductal adenocarcinoma cells L3.6, BxPC3 and Panc1	[67]
RNA-seq	SAHA	Patient-derived glioma stem cells	[68]
microarray	compound 106	brain, cerebellum, and heart of wild type and KIKI mice	[69]
microarray	MS275	mouse nucleus accumbens tissue	[70]
microarray	TSA, SAHA and VPA	mouse macrophages and dendritic cells	[71]
microarray	SAHA	rat dental pulp cells	[72]
microarray	2-ethyl-4-methylpentanoic acid, VPA and (S)-2-pentyl-4-pentynoic acid	undifferentiated R1 mouse embryonic stem cell	[73]
ChIP-seq and RNA-seq	SAHA	neuronal and non-neuronal cells of the hippocampal region and liver in mouse model	[74]
microarray	HDACi 4b	mouse brain and muscle tissue	[75]
microarray, small RNA-seq and miR-HTS	TSA	fibro-adipogenic progenitors from mdx mice	[39]
LC-MS/MS	SAHA	wild-type and patient NPC1^I1061T^ fibroblasts	[76]
SILAC and HSMS	FK228	esophageal squamous cell carcinoma EC109 cells	[77]
SILAC	SAHA and VPA	acute myeloid leukemia HL60 cells	[78]
MS acetylome	19 HDIs	HeLa cervical cancer cells	[79]
MS metabolomics	Butyrate and TSA	colorectal adenocarcinoma HT29 cells	[80]
NMR	compound i8, i10, i12, SAHA, TSA and nicotinamide	glioblastoma cell lines U373 and LN229	[81]
LC/GC-MS/MS	LBH589	plasma from diffuse large B-cell lymphomas (DLBCL) patients and DLBCL cell line OCI-Ly1 and OCI-Ly7	[82]
affinity capture > MS	16 HDIs	K562 myelogenous leukemia cells, Jurkat E6.1 cells, and Ramos lymphoma cells	[83]
affinity capture > MS	tacedinaline	K562 myelogenous leukemia cells	[84]
beads MS	SAHA	HeLa cervical cancer cells	[85]
multidimensional protein identification technology (MudPIT)	compound 106	Friedreich’s ataxia patient iPSC-derived neural stem cells	[86]
